# Evidence of recombination within human alpha-papillomavirus

**DOI:** 10.1186/1743-422X-4-33

**Published:** 2007-03-28

**Authors:** Manuel Angulo, Antonio Carvajal-Rodríguez

**Affiliations:** 1 Department of pathology. Complejo Hospitalario Universitario de Vigo, 36204 Vigo, Spain; 2 Departamento de Bioquímica, Genética e Inmunología. Universidad de Vigo, 36310 Vigo, Spain

## Abstract

**Background:**

Human papillomavirus (HPV) has a causal role in cervical cancer with almost half a million new cases occurring each year. Presence of the carcinogenic HPV is necessary for the development of the invasive carcinoma of the genital tract. Therefore, persistent infection with carcinogenic HPV causes virtually all cervical cancers. Some aspects of the molecular evolution of this virus, as the putative importance of recombination in its evolutionary history, are an opened current question. In addition, recombination could also be a significant issue nowadays since the frequency of co-infection with more than one HPV type is not a rare event and, thus, new recombinant types could be currently being generated.

**Results:**

We have used human alpha-PV sequences from the public database at Los Alamos National Laboratory to report evidence that recombination may exist in this virus. A model-based population genetic approach was used to infer the recombination signal from the HPV DNA sequences grouped attending to phylogenetic and epidemiological information, as well as to clinical manifestations. Our results agree with recently published ones that use a different methodology to detect recombination associated to the gene L2. In addition, we have detected significant recombination signal in the genes E6, E7, L2 and L1 at different groups, and importantly within the high-risk type HPV16. The method used has recently been shown to be one of the most powerful and reliable procedures to detect the recombination signal.

**Conclusion:**

We provide new support to the recent evidence of recombination in HPV. Additionally, we performed the recombination estimation assuming the best-fit model of nucleotide substitution and rate variation among sites, of the HPV DNA sequence sets. We found that the gene with recombination in most of the groups is L2 but the highest values were detected in L1 and E6. Gene E7 was recombinant only within the HPV16 type. The topic deserves further study because recombination is an important evolutionary mechanism that could have high impact both in pharmacogenomics (i.e. on the influence of genetic variation on the response to drugs) and for vaccine development.

## Background

Presence of the carcinogenic human papillomavirus (HPV) is necessary for the development of the invasive carcinoma of the genital tract [1]. Persistent infection with carcinogenic HPV causes virtually all cervical cancers. According to the latest global estimates this cancer is the second most common in women after breast cancer [2]. Almost half a million new cases of cervical cancer occur each year among women world-wide causing 274.000 deaths, 85% of them happening in underdevelopment countries[3].

The HPV genome has three different regions: two coding (E – early and L – late expression) and a regulatory non-coding region. The early region codifies regulatory, transforming and replication proteins, among which E6 and E7 are known to act like oncoproteins in high risk virus types [4,5]. The late region (L) contains two coding genes, L1 and L2, which encode viral capsid proteins.

Among more than 100 types of HPV known today, approximately 30 infect the genital tract. Within these, HPV16 and HPV18 are the two types with the highest oncogenic power. A prospective way of fighting cervical cancer is with an anti-HPV vaccine. Phase III vaccine trials are being developed by Merck, GlaxoSmithKline and the National Cancer Institute [5,6]. Besides, research is ongoing on different aspects of HPV biology, such as the mechanisms of down-regulation through which HPV causes cell transformation, the evaluation of biomarkers for risk progression, the role of environmental co-factors and the determinants of immune response to the viral infection [1]. However, to have a better understanding of the HPV, it is key to gain an improved insight from an evolutionary perspective [7,8]. The possibility for HPV recombination was first suggested by several facts: The biological viability of artificial HPV strains with chimerical proteins [9]. The appearance of some HPV16 variants that seemed mosaics between different established types [10]. The isolation of a novel HPV type (HPV77) with an unusual pattern of sequence similarity over the E6, E7 and L1 regions [11,12]. The plurality of HPV types [13] and also by the frequent observed co-infection (M. Angulo, personal observation). The latter will be specially important in AIDS patients which are often co-infected with very diverse mixtures of human papillomavirus (HPV) [14-17].

Because of HPV extreme diversity, the occurrence of recombination was initially discarded, in part because of the technical difficulties for aligning extremely diverse sequences, and in part because of the less accurate methods available for the researchers until the past decade. Nevertheless, recently reported phylogenetic incongruence at the putative high-risk ancestor node, showing that one or more presumed old recombination events should explain a non monophyletic evolution of oncogenic HPVs [18,19], has provided new convincing support of recombination in α-PVs. In addition, a recent rigorous analysis, using several recombination estimation methods, has provided fresh evidence of ancient recombination in papillomavirus, especially for the L2 gene [20].

However, the methods used to assess the presence of recombination signals were either phylogenetic based or substitution based. No model-based method was used. Hence, the difficulty of aligning all currently sequenced PVs imposes an additional challenge. Here we addressed the existence of recombination in HPV using a very efficient composite likelihood method [21,22]. The advantages of this kind of method, which is a model-based method, over the model-free ones, are well-known [23] including the fact that with model-free ones the true level of recombination that has occurred is greatly underestimated.

We have centered on human alpha PV sequences, which alignment is much more reliable. Our goal was to get recombination estimates of different genes (E6, E7, L1 and L2) in different groups. We defined three groups (GI, GII and GIII) attending to their phylogenetic relationships but also to their epidemiology and clinical outcome. The identification of the specific recombinant sequences or the recombination break points is a more complex problem and requires different algorithms and software, being out of the scope of the present paper.

## Results

### Evolutionary model and rate variation

Table 1 shows the best-fit models of nucleotide substitution selected for each data set. Different models were selected for the different genes and for the different groups. The simplest models were found within the HPV16 group and the most complex ones (GTR) for the gene L2 in GI and GII groups. L2 had also the most complex model within the HPV16 and the GIII groups (Table 1).

**Table 1:** Evolutionary models for the different data sets

Sequences	Nucleotide Model	Base Freqs	Rates	Invariable Sites	Rate Variation
HPV16_L1	HKY+I	A = 0.32	Ti/tv = 3.4	0.93	No
		C = 0.19			
		G = 0.19			
		T = 0.30			
HPV16_L2	TrN+I	A = 0.31	R(a) = 1.0	0.89	No
		C = 0.22	R(b) = 6.5		
		G = 0.16	R(c) = 1.0		
		T = 0.31	R(d) = 1.0		
			R(e) = 3.9		
			R(f) = 1.0		
HPV16_E6	HKY+I	A = 0.34	Ti/tv = 1.2	0.90	No
		C = 0.16			
		G = 0.22			
		T = 0.28			
HPV16_E7	HKY+I	A = 0.31	Ti/tv= 7.0	0.95	No
		C = 0.21			
		G = 0.23			
		T = 0.25			
GI_L1	TVM+I+G	A = 0.31	R(a) = 4.5	0.29	1.47
		C = 0.18	R(b) = 7.9		
		G = 0.19	R(c) = 3.4		
		T = 0.32	R(d) = 3.1		
			R(e) = 7.9		
			R(f) = 1.0		
GI_L2	GTR+I+G	A = 0.29	R(a) = 2.4	0.17	1.67
		C = 0.21	R(b) = 4.6		
		G = 0.18	R(c) = 2.8		
		T = 0.32	R(d) = 1.7		
			R(e) = 3.3		
			R(f) = 1.0		
GI_E6	TVM+I+G	A = 0.37	R(a) = 4.0	0.17	2.41
		C = 0.17	R(b) = 7.1		
		G = 0.21	R(c) = 2.8		
		T = 0.25	R(d) = 5.4		
			R(e) = 7.1		
			R(f) = 1.0		
GI_E7	HKY+G	A = 0.33	Ti/tv = 1.2	0.00	0.85
		C = 0.23			
		G = 0.22			
		T = 0.22			
GII_L1	TVM+G	A = 0.29	R(a) = 3.6	0.00	0.56
		C = 0.18	R(b) = 5.4		
		G = 0.22	R(c) = 2.3		
		T = 0.30	R(d) = 1.8		
			R(e) = 5.4		
			R(f) = 1.0		
GII_L2	GTR+G	A = 0.25	R(a) = 2.3	0.00	0.84
		C = 0.24	R(b) = 4.8		
		G = 0.20	R(c) = 2.6		
		T = 0.30	R(d) = 1.4		
			R(e) = 3.2		
			R(f) = 1.0		
GII_E6	TVM+G	A = 0.31	R(a) = 3.1	0.00	0.69
		C = 0.20	R(b) = 6.1		
		G = 0.23	R(c) = 2.2		
		T = 0.26	R(d) = 2.8		
			R(e) = 6.1		
			R(f) = 1.0		
GII_E7	TVM+G	A = 0.31	R(a) = 3.7	0.00	1.25
		C = 0.22	R(b) = 7.5		
		G = 0.24	R(c) = 3.9		
		T = 0.23	R(d) = 5.0		
			R(e) = 7.5		
			R(f) = 1.0		
GIII_L1	TVM+I+G	A = 0.24	R(a) = 2.7	0.29	0.83
		C = 0.23	R(b) = 4.8		
		G = 0.22	R(c) = 1.1		
		T = 0.30	R(d) = 0.9		
			R(e) = 4.8		
			R(f) = 1.0		
GIII_L2	TVM+I+G	A = 0.22	R(a) = 1.5	0.33	1.85
		C = 0.26	R(b) = 3.1		
		G = 0.22	R(c) = 1.7		
		T = 0.30	R(d) = 0.7		
			R(e) = 3.1		
			R(f) = 1.0		
GIII_E6	TrN+G	A = 0.32	R(a) = 1.0	0.00	0.68
		C = 0.22	R(b) = 2.2		
		G = 0.26	R(c) = 1.0		
		T = 0.20	R(d) = 1.0		
			R(e) = 4.0		
			R(f) = 1.0		
GIII_E7	HKY+I	A = 0.27	Ti/tv = 1.1	0.32	Equal
		C = 0.20			
		G = 0.30			
		T = 0.23			

I: Invariable sites. G: Rate variation. R(a):A-C. R(b): A-G. R(c): A-T. R(d): C-G. R(e): C-T. R(f): G-T. Ti/tv: Transition/transversion rate.

In addition, rate variation among sites has been detected in several of the data sets, though only in few ones the shape value of the gamma distribution was below one indicating an important rate heterogeneity [24]. The significant rate variation (below one) was mainly detected for GII and GIII groups (Table 1).

### Population recombination

**Figure 1:**
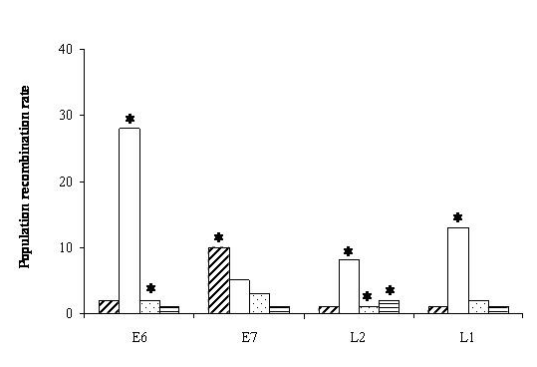
**HPV population recombination estimates under a gene conversion model assuming a Jukes-Cantor evolution model with 2 alleles**. For each gene and from left to right: Bars with upper right lines: HPV-16. Empty bars: GI. Light shaded bar: GII. Bars with horizontal lines: GIII. *: Recombination test was significant.

Using a gene conversion model with a Jukes-Cantor nucleotide evolution model [25] and assuming no rate variation we found significant recombination in all genes and in all groups (Figure 1). The highest value of recombination was found associated with E6 and the highest number of groups with recombination was linked to L2. Recombination was detected in the genes L1, L2 and E6 only for the GI group (high risk). For the gene E7 recombination was detected only within HPV16 type within which, however, no other recombinant gene were detected.

**Figure 2:**
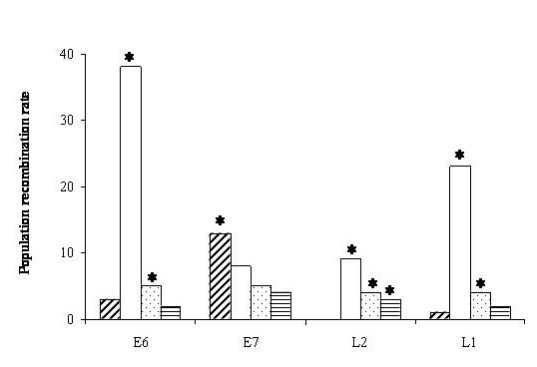
**HPV population recombination estimates under a gene conversion model with complex substitution models and rate variation among sites**. For each gene and from left to right: Bars with upper right lines: HPV-16. Empty bars: GI. Light shaded bar: GII. Bars with horizontal lines: GIII. *: Recombination test was significant.

When using the best-fit models of nucleotide substitution and considering the estimated rate variation the obtained results were qualitatively similar but with a bit higher estimates and a new signal for L1 in the GII group (cf. Figures 1 and 2).

Given the estimated recombination values and the number of sequences at each group, the expected number of recombination events associated with each data set can be computed [26]. For example, for HPV-16 with a recombination value of 13 for the gene E7 (Figure 2) and *n *= 8 sequences, the expected total number of recombination events in the history of this sample is 34. The expected numbers of recombination events for the different data sets with detected significant recombination are shown in Table 2.

**Table 2:** Expected number of recombination events

Gene	Group	Number of sequences	Recombination value	Recombination Events
L1	I	14	23	73
L1	II	8	4	10
L2	I	14	9	29
L2	II	8	4	10
L2	III	12	3	9
E6	I	14	38	121
E6	II	8	5	13
E7	HPV16	8	13	34

### Simulation experiment

A set of simulations was performed using the parameter values corresponding to the evolutionary model, base composition and rate variation among sites for the E6 gene in the group GI (Table 1) to obtain some control samples of DNA sequences. This gene and group were selected because this combination had the highest estimated significant value (Figures 1 and 2 and Table 2). We set the recombination value to zero so that the simulated set of sequences was obtained without recombination. We then estimated from these sequences the population recombination value using the composite likelihood. The average recombination value for the simulated set of sequences was 0.08 ± 0.03 and the percentage of false positive recombination tests was 1%. The expected number of recombination events for this average number is 0.26.

## Discussion

The estimation of the best-fit model of nucleotide substitution is relevant in phylogenetics [27]. However, model-based approaches for estimating recombination do not rely on a specific phylogeny and in consequence, they are expected to be robust to model misspecification. This seems to be the case when estimating recombination using the composite likelihood [21,22]. Nevertheless, we have provided the best-fit models of nucleotide substitution under the Akaike information criteria (AIC) and have used them to estimate the population recombination rate. As expected, the model complexity had not a major effect on the estimation.

The existence of the recombination signal in the DNA sequences of HPVs is important because the genes tested are commonly used to build HPV phylogenies [28] and it is known that recombination can mislead phylogenetic inferences [29]. Furthermore, the detected recombination is in agreement with recently reported phylogenetic incongruence at the putative high-risk ancestor node showing that one or more presumed old recombination events should explain a non monophyletic evolution of oncogenic HPVs [18,19]. The confirmation of ancient papillomavirus recombination has also been recently thoroughly argued by a statistical and phylogenetic recombination detection study [20].

In contrast to that previous study, we have used a model-based method (the coalescent composite likelihood estimator) to infer recombination from HPV DNA sequences. Model-based methods are known to be preferred over substitution and phylogenetic ones [23,30-32]. Thus, the composite-likelihood estimator maximizes the chances of detecting recombination avoiding, however, the inference of recombination when it is absent (false positive detection).

Regarding the existence of model complexity and rate variation among sites in the HPV samples, it is known that the amount of divergence and rate variation in the data could affect recombination estimation in some cases [32]. However, rate variation i.e. variation in the rate of nucleotide substitution along the sites in the DNA sequence should have no effect on the recombination estimates obtained using the composite-likelihood estimator as has been previously shown [22]. However, the *Pairwise *program assumes a simple Jukes-Cantor model with two alleles [21]. Therefore, to account for possible effects of model complexity and rate variation we also estimated the recombination rate using the extended model possibilities of *Kpairwise *[22] and confirmed that model complexity and rate variation had no qualitative effect onto recombination estimates.

Moreover, we have designed an experiment to check the possibility of false positive detection due to recombination artifacts because of the model complexity and the high diversity underlying the data. As shown above, there was not significant recombination estimation under the parameters considered.

In addition, we were able to estimate the expected number of recombination events in those cases with significant recombination detection. As expected, the higher numbers were found for group GI, which incorporates a major number of branches from PV phylogeny [19]. Importantly, not all recombination events are detectable [32], thus those expectations just provide an upper-bound below which the real number of detectable events should relay [30].

Although we have detected significant recombination signal at all genes and groups in one combination or another, perhaps the most important result is that recombination was detected at intra-type level in HPV16. This may indicate that recombination is occurring at a relative high frequency in current carcinogenic HPV types and variants. HPV recombination should not be exceptional nowadays since the frequency of co-infection with more than one HPV type is not a rare event, and new recombinant types could be currently being generated. Provided that the oncogenicity of specific HPV intra-type variants appear to vary geographically and also with the ethnic origin of the population studied [33], more research is necessary to assess whether such a variation could relate to different recombinant forms. Moreover, the majority of the vaccines under investigation, both the therapeutic and the prophylactic ones, are based on the use of these genes or their products, to obtain the prevention and the treatment of the infections of the more prevalent high risk (types 16 and 18) and low risk types (6 and 11) [6/and references there in, 34/and references there in]. From the present work it is clear that a better knowledge of HPV evolutionary relationships will be important concerning the optimal number of types to include in vaccines as well as the possibility of cross-reactive immunity among HPV types [2,5]. Also, to obtain consensus and ancestor HPV sequences, this could be used in vaccine design to minimize genetic differences between vaccine strains [35]. The existence of recombination should also be of interest to pharmacogenomic studies, i.e. to learn how genetic variation influence response to drugs.

## Conclusion

We provide new support to the recent evidence of recombination in HPV. In addition, we perform an evolutionary characterization, estimating best-fit models of nucleotide substitution and rate variation among sites, of some important HPV DNA sequence sets. Using simulations, we have shown that the detected recombination signals should not be artifacts. Thus, we found that the gene with recombination in most of the groups is L2 but the highest values were detected in L1 and E6. Gene E7 was recombinant only within the HPV16 type. The topic deserves further study because recombination is an important evolutionary mechanism that could have high impact in both pharmacogenomics (i.e. the effect of genetic variation on response to drugs) and vaccine development.

## Methods

### Sequences, groups and models of nucleotide substitution

HPV sequences for the genes E6, E7, L1 and L2 were obtained from the public database at Los Alamos National Laboratory [36] (GenBank accession numbers are given in Table 3). We classified these sequences according to phylogenetic criteria [19] but also by epidemiological criteria and clinical outcome [37]. The groups set as follows: Group I (GI) included the 14 most common high-risk types (16, 18, 31, 33, 35, 39, 45, 51, 52, 56, 58, 59, 73, 82; *n *= 14 sequences including just one variant of type 16). Group II (GII) included 6 low risk types (6, 11, 40, 42, 43, 44; *n *= 8 sequences including the 3 variants of type 6, see Table 3). Group III (GIII) included 3 low risk types plus 5 undetermined risk types which cluster together [19] (61, 72, 81, 62, 71, 83, 84, 89; *n *= 12 or 11 sequences including 5 or 4 variants of type 71, see Table 3. For this group, L1 has 13 sequences because of 4 variants from type 71 plus 2 additional variants from 72 and 81 types). Finally, we consider *per se *the group *HPV-16*, which included HPV16 variants (*n *= 8 sequences, Table 3). All selected sequences pertain to the genus Alpha [38]. Sequences were aligned with ClustalX [39] and then corrected by hand. The best-fit model of nucleotide substitution was selected under the Akaike information criteria (AIC) with Modeltest v3.6 [40], using maximum likelihood (ML) estimates from PAUP* [41].

**Table 3:** Accession numbers for HPV sequences including genes E6, E7, L2 and L1

**Group I**	**Accession number**	**Group II**	**Accession number**
HPV 16	[GenBank:NC_001526,	HPV 6	[GenBank:NC_000904,
	GenBank:AF125673,		GenBank:NC_001355,
	GenBank:AF402678,		GenBank:NC_001668]
	GenBank:AF472508,		
	GenBank:AF472509,		
	GenBank:AF534061,		
	GenBank:AF536179,		
	GenBank:AF536180]		
HPV 18	[GenBank:NC_001357]	HPV 11	[GenBank:NC_001525]
HPV 31	[GenBank:NC_001527]	HPV 40	[GenBank:NC_001589]
HPV 33	[GenBank:NC_001528]	HPV 42	[GenBank:NC_001534]
HPV 35	[GenBank:NC_001529]	HPV 43	[GenBank:NC_005349]
HPV 39	[GenBank:NC_001535]	HPV 44	[GenBank:NC_001689]
HPV 45	[GenBank:NC_001590]	**Group III**	
HPV 51	[GenBank:NC_001533]	HPV 61	[GenBank:NC_001694]
HPV 52	[GenBank:NC_001592]	HPV 72	[GenBank:NC_006164]
HPV 56	[GenBank:NC_001594]	HPV 81	[GenBank:NC_005351]
HPV 58	[GenBank:NC_001443]	HPV 62	[GenBank:NC_006357]
HPV 59	[GenBank:NC_001635]	HPV 71	[GenBank:AY330620,
			GenBank:AY330621,
			GenBank:AY330622,
			GenBank:AY330623,
			GenBank:NC_002644]
HPV 73	[GenBank:NC_006165]	HPV 83	[GenBank:NC_000856]
HPV 82	[GenBank:NC_002172]	HPV 84	[GenBank:NC_002676]
		HPV 89	[GenBank:NC_004103]

### Recombination estimation

To study HPV recombination we used the composite likelihood estimator [42] and its permutation test [21], which is one of the most powerful techniques to detect the recombination signal from DNA sequences [22]. This method is a model-based population genetic approach, which allows for both a linear recombination and a gene conversion model. The composite likelihood estimator is implemented by the program *Pairwise *from the package Ldhat, freely available at [43], and also by the extension *Kpairwise*[22] which allows for complex nucleotide models and rate variation among sites and is freely available at [44]. We considered using a gene conversion model as more adequate since the PV genome is circular. Recombination was estimated as the population recombination rate, which is *4Nr *where *N *is the effective population size and *r *the recombination rate per gene.

Given the recombination values estimated and the number of sequences at each group the expected number of recombination events *E*(*R*) associated to each data set can be computed by using formulae 5 in Hudson and Kaplan [26],

E(R)=(4Nr)∑i=1n−11i
 MathType@MTEF@5@5@+=feaafiart1ev1aaatCvAUfKttLearuWrP9MDH5MBPbIqV92AaeXatLxBI9gBaebbnrfifHhDYfgasaacH8akY=wiFfYdH8Gipec8Eeeu0xXdbba9frFj0=OqFfea0dXdd9vqai=hGuQ8kuc9pgc9s8qqaq=dirpe0xb9q8qiLsFr0=vr0=vr0dc8meaabaqaciaacaGaaeqabaqabeGadaaakeaacqWGfbqrcqGGOaakcqWGsbGucqGGPaqkcqGH9aqpcqGGOaakcqaI0aancqWGobGtcqWGYbGCcqGGPaqkdaaeWbqaamaalaaabaGaeGymaedabaGaemyAaKgaaaWcbaGaemyAaKMaeyypa0JaeGymaedabaGaemOBa4MaeyOeI0IaeGymaedaniabggHiLdaaaa@420E@, where *n *is the number of sequences in the sample.

### Simulations

To check that HPV sequences do not generate recombination false positives under the composite likelihood estimator due to their particular combination of model complexity and rate variation, we simulated 100 DNA samples of 15 sequences each and longitude 500 bp using a coalescent model [45]. We used the software *recoal1.7 *from David Posada and available upon request from him [46]. Specifically, we set the simulations with the parameter values corresponding to the evolutionary model, base composition and rate variation for the gene and group which had the highest estimated significant value. We also set the recombination value to zero. We then used the obtained sequences to estimate the average recombination value and the percentage of false positive recombination tests.

## Competing interests

The author(s) declare that they have no competing interests.

## Authors' contributions

MA carried out the acquisition of data, sequence alignment, participated in recombination analyses and helped to draft the manuscript. AC-R conceived and designed the study, participated in the recombination analyses, performed data analysis and simulations and drafted the manuscript. All authors read and approved the final manuscript.
